# Identification of Inflammatory Markers for the Prediction and Diagnosis of Diminished Ovarian Reserve Using Olink Targeted Proteomics

**DOI:** 10.3390/jcm15114072

**Published:** 2026-05-25

**Authors:** Meihui Li, Yu Zhang, Lin Yu, Yan Shi, Minzhi Gao, Nian Huang, Zhaogui Sun

**Affiliations:** 1 Shanghai-MOST Key Laboratory of Health and Disease Genomics, NHC Key Lab of Reproduction Regulation, Shanghai Institute for Biomedical and Pharmaceutical Technologies, Clinical Medical School, Fudan University, Shanghai 200032, China; 2 Shanghai Key Laboratory for Assisted Reproduction and Reproductive Genetics, Center for Reproductive Medicine, Renji Hospital, School of Medicine, Shanghai Jiaotong University, Shanghai 200135, China; 3 Department of Integrative Medicine, Eastern Hepatobiliary Surgery Hospital, Naval Medical University, Shanghai 200438, China

**Keywords:** diminished ovarian reserve, follicular fluid, Olink proteomics, inflammation, diagnostic index

## Abstract

**Objectives:** Diminished ovarian reserve (DOR) significantly compromises in vitro fertilization (IVF) success. Although systemic markers such as anti-Müllerian hormone (AMH) serve as valuable clinical indicators of the ovarian reserve, they lack the sensitivity to reflect the qualitative deterioration of the follicular microenvironment. Therefore, in this study, we aimed to characterize the inflammatory proteome of follicular fluid (FF) to establish a high-performance auxiliary diagnostic model for DOR. **Methods:** Utilizing the ultra-sensitive Olink proximity extension assay, we quantified 92 inflammation-related proteins in the FF of 88 participants (67 with DOR and 21 normal controls). Differentially expressed proteins (DEPs) were identified, and their relationships with key clinical indices were evaluated. A robust predictive signature was refined through integrated Least Absolute Shrinkage and Selection Operator (LASSO) regression and Random Forest algorithms, with diagnostic performance assessed via 10-fold cross-validation. **Results:** Thirty-five DEPs were significantly dysregulated in the FF of patients with DOR, demonstrating strong associations with serum AMH and basal estradiol concentrations. A minimized diagnostic panel comprising four core proteins, adenosine deaminase (ADA), vascular endothelial growth factor A (VEGFA), eukaryotic translation initiation factor 4E-binding protein 1 (4E-BP1), and matrix metalloproteinase-1 (MMP-1), was established. This multivariable model achieved an excellent area under the receiver operating characteristic curve (AUC) of 0.953. **Conclusions:** The identified four-protein signature reflects localized chronic inflammation and early pathophysiological shifts in the DOR follicular microenvironment. As a high-performance molecular index, this panel could complement conventional systemic assessments, provide a reliable means of evaluating follicular viability, and optimize individualized therapeutic strategies.

## 1. Introduction

Diminished ovarian reserve (DOR) is a formidable challenge in contemporary reproductive medicine and is characterized by a decline in both the quantity and quality of oocytes [1,2]. Clinically, DOR severely compromises the outcomes of in vitro fertilization (IVF), resulting in higher cycle cancellation rates, the retrieval of fewer oocytes, poor embryo quality, and a higher risk of early miscarriage [3,4,5]. The effective management of the clinical risks of DOR is crucial to improve reproductive prognoses.

Currently, the diagnosis of DOR follows the 2012 guidelines of the American Society for Reproductive Medicine Practice Committee; there is no universally accepted definition of DOR [6]. In clinical practice, the diagnosis of DOR predominantly relies on the use of systemic endocrine markers, primarily the serum anti-Müllerian hormone (AMH) concentration and the antral follicle count (AFC) [7,8]. Although these parameters accurately reflect declines in the size of the follicle pool, they fail to capture qualitative deterioration of the ovarian microenvironment, which directly dictates oocyte viability and maturation [9,10]. This diagnostic gap highlights the urgent need for the identification of novel, local biomarkers that would complement conventional systemic evaluations.

The follicular fluid (FF), which directly nurtures the oocyte, provides an ideal window into the local microenvironment of oocytes [11,12]. Emerging evidence indicates that “inflammaging,” a state of chronic, low-grade inflammation, is a primary driver of DOR [13]. Inflammaging, one of the crucial downstream mechanisms of ovarian aging, is characterized by progressive and sustained systemic proinflammatory stress [14]. It involves disruption of immune tolerance, impairment of angiogenesis, and the activation of granulosa cell apoptosis [13]. In patients with DOR, elevated intrafollicular concentrations of IL-6 and IL-8 are significantly associated with increased total oxidant status and oxidative stress index [15]. This localized inflammatory and oxidative imbalance compromises oocyte quality, resulting in diminished fertilization rates and impaired embryo development [15]. Intrafollicular inflammatory factors serve as a primary determinant of IVF outcomes, highlighting how an altered microenvironment impairs reproductive potential. Consequently, profiling the secreted proinflammatory proteins within the FF represents a highly feasible means of identifying auxiliary diagnostic criteria and novel therapeutic targets [16].

Currently, there is no unified set of diagnostic criteria for DOR, and the precise effects of follicular inflammation on reproductive outcomes have been poorly characterized. Historically, efforts to comprehensively profile this microenvironment were hindered by the extremely low abundance of intrafollicular cytokines. In the present study, to overcome these clinical and analytical barriers, we employed the ultra-sensitive Olink proximity extension assay (PEA) and machine learning algorithms to systematically map the inflammatory landscape of FF in patients with DOR. Through this approach, we aimed to construct a robust, multi-protein diagnostic model, elucidate the mechanisms underlying follicular inflammaging, and provide a novel, high-precision tool for the early diagnosis of DOR and the clinical evaluation of the follicular microenvironment.

## 2. Materials and Methods

### 2.1. Participants and Ethics Approval

We recruited 88 women (21 in the control group and 67 in the DOR group) who underwent IVF, including intracytoplasmic sperm injection (ICSI) and preimplantation genetic testing for aneuploidy (PGT-A), at Renji Hospital, Shanghai Jiao Tong University School of Medicine, between January 2024 and April 2024. Baseline demographic and clinical characteristics of all study participants are summarized in Supplementary Table S1. The study protocol was approved by the Shanghai Institute of Planned Parenthood Research (approval no. PJ2020-12) and conducted in accordance with the principles of the Declaration of Helsinki. All the participants provided their written informed consent prior to enrollment.

The inclusion criteria for the study were as follows. For the DOR group, age ≤ 40 years and one of the following three criteria: (1) AMH concentration < 1.1 ng/mL, (2) AFC ≤ 7 follicles in both ovaries, or (3) basal follicle-stimulating hormone (FSH) concentration ≥10 IU/L for two consecutive menstrual cycles. For the Control group (CON), age ≤ 40 years; the presence of regular menstrual cycles (25–35 days) with confirmed ovulation; a diagnosis of infertility; one of the following two criteria: (1) tubal patency test indicating tubal obstruction, hydrosalpinx, or a history of tubal surgery; or (2) male factor infertility, diagnosed in accordance with the sixth edition of the WHO Laboratory Manual for the Examination and Processing of Human Semen (2021); and the presence of no other causes of infertility, such as ovulatory disorders or uterine abnormalities. The exclusion criteria were comorbidities, including thyroid dysfunction or adrenal disease; hyperprolactinemia or a neurological disorder; a chromosomal abnormality; a body mass index (BMI) <18 or >28 kg/m^2^; and contraindication for IVF and embryo transfer.

### 2.2. Follicular Fluid Collection

All the participants underwent controlled ovarian hyperstimulation according to a standard protocol at Renji Hospital. During oocyte retrieval, FF was collected from follicles with a diameter of 16–20 mm using sterile aspiration needles (Cook Medical Holdings LLC, Daniels Way, Bloomington, IN, USA). Following aspiration, the oocyte–cumulus complexes were immediately isolated from the fluid under a stereomicroscope by an embryologist for subsequent IVF/ICSI. The remaining oocyte-free FF samples were then centrifuged at 500× *g* for 10 min at 4 °C to remove residual cellular debris before being aliquoted and stored at −80 °C.

### 2.3. Screening for Inflammatory Biomarkers Using Olink

The Olink Target 96 Inflammation Panel (Olink Proteomics AB, Uppsala, Sweden) was used to quantify the expression of 92 inflammation-related proteins, according to the manufacturer’s protocol. Briefly, 10 μL of each FF sample was added to a pre-coated 96-well plate containing paired antibody probes conjugated to unique DNA oligonucleotides associated with barcodes, and then PEA was performed. When the paired antibodies bound to the target protein, the adjacent DNA barcodes were extended by DNA polymerase, and the resulting amplicons were quantified using real-time polymerase chain reaction assays.

Olink targeted proteomics data are expressed as log2-scaled normalized protein expression (NPX) values. The raw data were subjected to inter-plate control (IPC) normalization to mitigate batch effects. Missing data were handled by performing imputation using the DMwR2 package in R (R Foundation for Statistical Computing, Vienna, Austria). Strict quality control was performed to exclude samples deviating by >0.3 × NPX from the plate median value and proteins with a detection rate <75% across all samples. This stringent filtering approach ensured that downstream analyses relied solely on robustly quantifiable targets without the need for missing value imputation. Differentially expressed proteins (DEPs) were subsequently identified using the R package ‘OlinkAnalyze’ (v.2.0), using *p* < 0.05 and an absolute fold change (|FC|) ≥1.2 to adequately balance the need for statistical stringency with the biological sensitivity required for the assessment of low-abundance follicular cytokines.

### 2.4. Biomarker Selection Using LASSO Regression and Random Forest

The R package “glmnet” (v.4.1-8) was used to perform LASSO regression with 10-fold cross-validation to identify potential biomarkers from among the DEPs. The optimal λ value was determined by minimizing the cross-validation error, and proteins with non-zero coefficients were selected as candidate biomarkers. The R package “randomForest” (v.4.7-1.1) was employed to construct a Random Forest (RF) model for biomarker selection. The number of trees was set to 500, and the importance of each protein was evaluated using the Gini coefficient. Proteins with a Gini coefficient >0.1 were considered to be highly important biomarkers. The proteins that were selected by both the LASSO regression and RF were defined as core biomarkers and were further analyzed.

### 2.5. Functional Enrichment Analysis

Gene Ontology (GO) and Kyoto Encyclopedia of Genes and Genomes (KEGG) pathway enrichment analyses were performed for the DEPs using the R package “clusterProfiler” (v.4.6-2). The GO analysis included the biological process, cellular component, and molecular function categories. The KEGG analysis focused on signaling pathways related to inflammation and reproductive physiology. Statistical significance was accepted at an adjusted *p*-value of <0.05. A protein–protein interaction (PPI) network was constructed using the STRING database (v.11.5) and visualized using Cytoscape (v3.10.2), to explore the interactions between the core biomarkers.

### 2.6. Evaluation of Diagnostic Performance Using Machine Learning

Three machine learning models (logistic regression (LR), support vector machine (SVM), and RF) were constructed to evaluate the diagnostic performance of the core biomarkers. The dataset was randomly split into a training set (70%) and a testing set (30%) using stratified sampling to maintain balance between the groups. The LR model was implemented with the R package “glm” (v.4.3.3). Model formula: auc_value <- auc(roc_obj, algorithm = 1). The SVM model was implemented with the R package “e1071” (v 1.7-13) using a radial basis function kernel, and the RF model was implemented as described in Section 2.4.

Model performance was evaluated using a receiver operating characteristic (ROC) curve, the area under the ROC curve (AUC), sensitivity, specificity, the positive predictive value, and the negative predictive value. The optimal cutoff value was determined using the Youden index (sensitivity + specificity − 1). Calibration curves and Hosmer–Lemeshow tests were used to assess model calibration. The robustness of the model was evaluated using 10-fold cross-validation, using nine data subsets iteratively for training and one for independent testing. Overfitting was assessed by comparing performance metrics, including the AUC, sensitivity, and specificity, of the training and testing phases across all the instances of cross-validation.

### 2.7. Statistical Analysis

Continuous variables are presented as mean ± standard deviation or median (interquartile range, IQR) if the data were normally or non-normally distributed, respectively, according to the Shapiro–Wilk test. Between-group comparisons were performed using Student’s *t*-test (normally distributed data) or the Mann–Whitney U test (non-normally distributed data). Categorical variables are presented as counts (percentages), and datasets were compared using the χ^2^ test or Fisher’s exact test. To quantify the strength of the associations between the groups, odds ratios and the corresponding 95% confidence intervals were calculated. Multivariate analysis was performed using binary logistic regression, and the results are also expressed as odds ratios and 95% confidence intervals. All the statistical analyses were performed using R software (v.4.3.3), and a two-tailed *p* < 0.05 was considered to indicate statistical significance.

## 3. Results

### 3.1. Characteristics of the Groups of Patients

FF samples obtained from women with a normal ovarian reserve (CON) or DOR were used for an Olink inflammatory proteomics analysis targeting ovarian reserve function. We studied FF samples from 88 individuals (21 samples from the Control group and 67 samples from the DOR group). After quality control performed through principal components analysis (PCA), 88 samples remained for analysis.

As shown in Table 1, the DOR group had distinct baseline characteristics and showed marked impairments in parameters reflecting ovarian reserve. The patients in the DOR group were significantly older (34.36 ± 3.55 vs. 29.62 ± 3.68 years, *p* < 0.001) and required a higher number of treatment cycles (3.31 ± 2.66 vs. 1.24 ± 0.63, *p* = 0.001). As anticipated, the ovarian reserve of the DOR group was markedly impaired, as shown by significantly lower serum AMH levels (0.97 ± 0.46 vs. 3.91 ± 1.17 ng/mL, *p* < 0.001) and AFC (4.79 ± 1.69 vs. 11.52 ± 4.21, *p* < 0.001). Conversely, BMI and baseline levels of FSH, LH, E2, T, and TSH showed no significant differences (all *p* > 0.05) between DOR and CON groups.

Regarding reproductive outcomes, the DOR group demonstrated a poorer prognosis: they had significantly fewer normally fertilized oocytes (10.61 ± 8.01 vs. 16.43 ± 7.50, *p* = 0.005) and transferable embryos (3.69 ± 2.17 vs. 5.62 ± 2.22, *p* = 0.001). Consequently, the clinical pregnancy rate of patients with DOR was substantially lower than that of CON (35.82% vs. 85.71%, *p* < 0.001). Additionally, a significant difference was observed in the distribution of ART methods used between the groups (*p* < 0.001).

### 3.2. Potential Inflammation-Related Biomarkers of DOR

**Figure 1:**
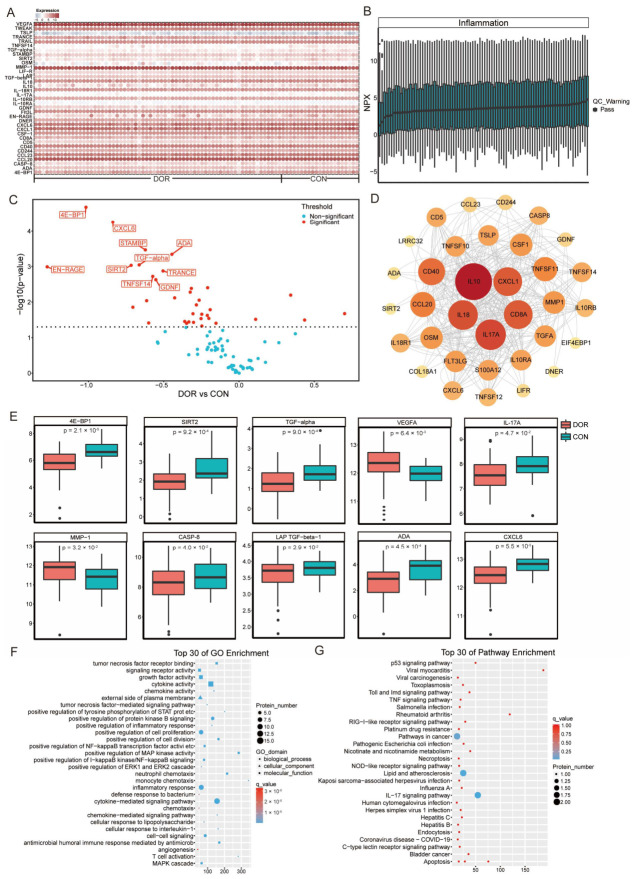
Distinct inflammation-related proteomic profiles were observed between DOR and CON groups. (**A**) Heatmap of the differentially expressed proteins (DEPs) in follicular fluid (red indicates upregulated expression; blue indicates downregulated expression). (**B**) Distribution of the normalized protein expression (NPX) values across samples. (**C**) Volcano plot illustrating the DEPs. (**D**) Protein–protein interaction (PPI) network. The node color intensity indicates the magnitude of the inter-group differences, and the node size represents the degree of connectivity. (**E**) Box plots of the 10 most significant DEPs. The center line indicates the median, and the box limits represent the interquartile range (IQR). Results of the (**F**) Gene Ontology (GO) and (**G**) Kyoto Encyclopedia of Genes and Genomes (KEGG) enrichment analyses of the DEPs.

Using the Olink Target 96 Inflammation Panel, we compared the expression levels of 92 inflammation-related proteins in FF samples from participants in the DOR and CON groups. A total of 35 inflammation-associated proteins were found to be differentially expressed, of which 32 proinflammatory proteins were upregulated, and three anti-inflammatory proteins were downregulated (Table 2, Figure 1A, Supplementary Table S2). All the samples passed QC (Figure 1B).

The DEPs are presented in a volcano plot in Figure 1C. Notably, the proteins that were significantly downregulated in the DOR group included 4E-BP, CXCL6, STAMBP, TGFα, ADA, EN-RAGE, SIRT2, TRANCE, TNFSF14, and GDNF. Because PPIs form the basis of cellular function and their disruption is often linked to disease pathogenesis, we constructed a PPI network to explore potential interactions among the DEPs. Within this network, there were high interaction scores for IL10, IL18, IL17A, CXCL1, and CD8A, suggesting that inflammation-related proteins play a key role in DOR (Figure 1D). The expression levels of the top 10 DEPs are shown as box plots in Figure 1E.

GO and KEGG pathway enrichment analyses were performed for the DEPs (Figure 1F,G). GO analysis indicated that these proteins are primarily involved in biological processes such as inflammatory responses and cytokine-mediated signaling (Figure 1F). KEGG enrichment analysis revealed that lipid metabolism pathways were significantly downregulated in the DOR group, whereas inflammatory pathways such as IL-17 and TNF-α signaling were markedly upregulated (Figure 1G). These differences were statistically significant, and therefore lipid metabolism pathways and IL-17-related inflammatory pathways may play important roles in the progression of DOR, and the secreted factors involved may represent biomarkers for the condition.

### 3.3. Relationships Between Clinical Features and Pregnancy Outcomes

**Figure 2:**
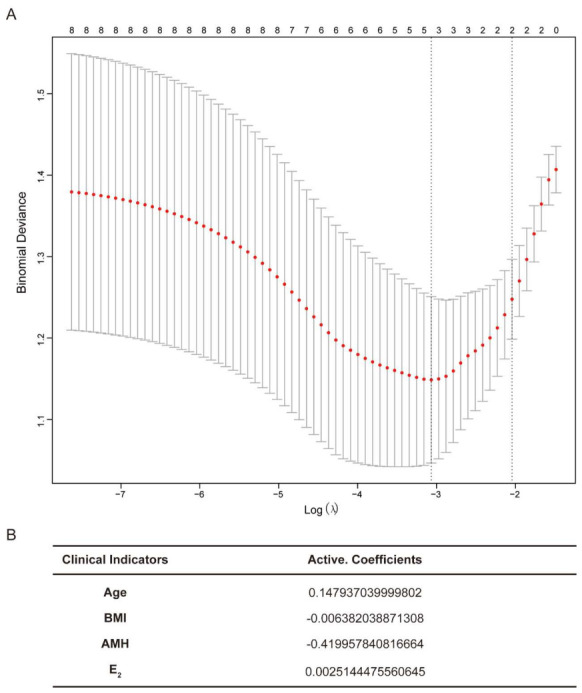
Relationships between pregnancy status and the clinical features of the participants. (**A**) Results of the LASSO regression analysis, showing the selection of four indices (age, BMI, AMH, and AFC) significantly correlated with pregnancy status. (**B**) Correlation coefficients for the relationships among the four clinical indices.

LASSO regression analysis was performed to evaluate the relationships between the clinical characteristics and pregnancy outcomes of the DOR and CON groups. The age, BMI, and AMH and E_2_ concentrations of the patients strongly correlated with their pregnancy outcomes (Figure 2A,B).

A correlation analysis was performed to evaluate the relationships between the clinical characteristics and pregnancy outcomes in the 67 patients with DOR. As shown in Table 3, the participants were divided into pregnant and non-pregnant groups according to their treatment outcomes. Univariate analysis was performed with respect to age, BMI, hormone concentrations, AFC, ovarian stimulation protocol, cycle number, number of oocytes retrieved, embryo number, and transfer timing. This revealed significant differences between the pregnant and non-pregnant groups with respect to age, the serum E_2_ concentration, and cycle number. These three variables were subsequently included in multivariate analysis, in which all three significantly differed between the groups (Table 3). These findings suggest that advanced age and low serum E_2_ concentrations may be associated with non-pregnancy outcomes.

### 3.4. Correlations Between DEP Levels and Serum Biomarker Concentrations

To investigate the relationships between the peripheral blood concentrations of E_2_, AMH, FSH, LH, TSH, testosterone, and the inflammatory status of FF, restricted cubic spline (RCS) models were employed to analyze the relationships between the DEPs and clinical indices in the participants with DOR and CON.

**Figure 3:**
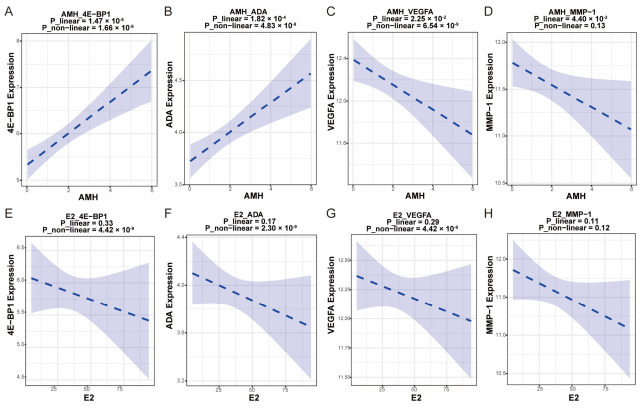
Restricted cubic spline (RCS) models illustrating the relationships between follicular fluid DEPs and clinical indices. (**A**–**D**) Associations of AMH concentrations with (**A**) 4E-BP1, (**B**) ADA, (**C**) VEGFA, and (**D**) MMP-1. (**E**–**H**) Associations of E2 concentrations with (**E**) 4E-BP1, (**F**) ADA, (**G**) VEGFA, and (**H**) MMP-1. The heavy blue dashed lines represent the estimated dose–response association, and the light purple-shaded areas denote the 95% confidence intervals (CIs). The exact *p*-values for both linear and non-linear trends are presented at the top of each respective panel.

As shown in Figure 3 and Supplementary Figure S1, we evaluated the relationships between the concentrations of FF proteins and key hormones. The AMH concentration was significantly associated with those of four target proteins (4E-BP1, ADA, VEGFA, and MMP-1), demonstrating both linear and non-linear patterns (Figure 3A–D). Similarly, E2 and FSH concentrations demonstrated predominantly non-linear associations with these proteins, and particularly with 4E-BP1 and VEGFA (Figure 3E–H, Supplementary Figure S1A–D). TSH showed complex associations with all four proteins (Supplementary Figure S1F–I), and LH demonstrated only a non-linear association with MMP-1 (Supplementary Figure S1E). The statistical parameters for both linear and non-linear models are provided in the corresponding figures.

### 3.5. Significant Diagnostic Values for the DEPs

**Figure 4:**
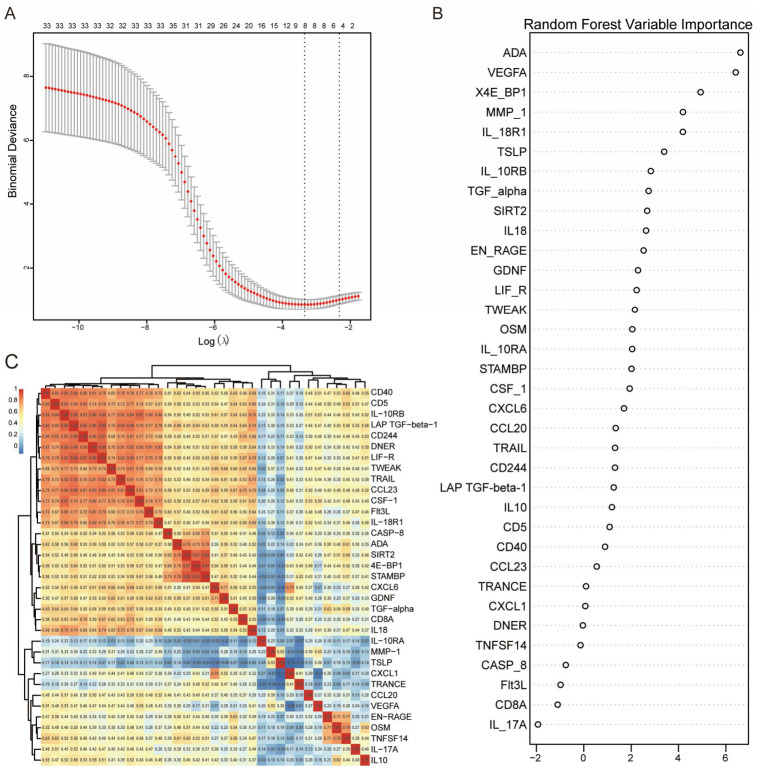
Identification of candidate diagnostic protein biomarkers for DOR. (**A**) Feature selection via LASSO logistic regression to identify the optimal diagnostic signature. (**B**) Key predictive proteins identified by Random Forest mean decrease accuracy. (**C**) Spearman correlation heatmap of the 35 DEPs (red indicates positive correlation; blue indicates negative correlation).

We employed LASSO regression analysis to further evaluate potential protein biomarkers in FF for use in the diagnosis of DOR and to evaluate the relationships between DEPs and the onset of DOR (Figure 4A). An RF model was used to calculate the variable importance of the DEPs, and the following top 10 protein biomarkers with the highest mean decrease accuracy (MDA) scores were identified: ADA (MDA: 6.61), VEGFA (MDA: 6.41), 4E-BP1 (MDA: 4.93), MMP-1 (MDA: 4.19), IL-18R1 (MDA: 4.19), TSLP (MDA: 3.39), IL-10RB (MDA: 2.83), TGF-alpha (MDA: 2.74), SIRT2 (MDA: 2.68), and IL18 (MDA: 2.63) (Figure 4B).

A heatmap illustrating the correlations among the 35 DEPs is shown in Figure 4C. Proteins associated with inflammatory function, including CD40, CD5, IL-10RB, LAP TGF-beta-1, CD244, DNER, LIF-R, TWEAK, CCL23, CSF-1, FLT3L, IL-18R1, and TRAIL, significantly correlated in the present sample set. In addition, proteins involved in cell proliferation, such as CASP-8, ADA, SIRT2, 4EBP1, and STAMBP, also exhibited significant correlations. These findings suggest that distinct changes in the concentrations of extracellular proteins in FF, and particularly those linked to inflammation and cell proliferation, may play a role in the pathogenesis of DOR.

Through the variable importance ranking of the RF and LASSO models described above, four core targets that were closely related to the incidence of DOR were ultimately identified: ADA, VEGFA, 4E-BP1, and MMP-1.

**Figure 5:**
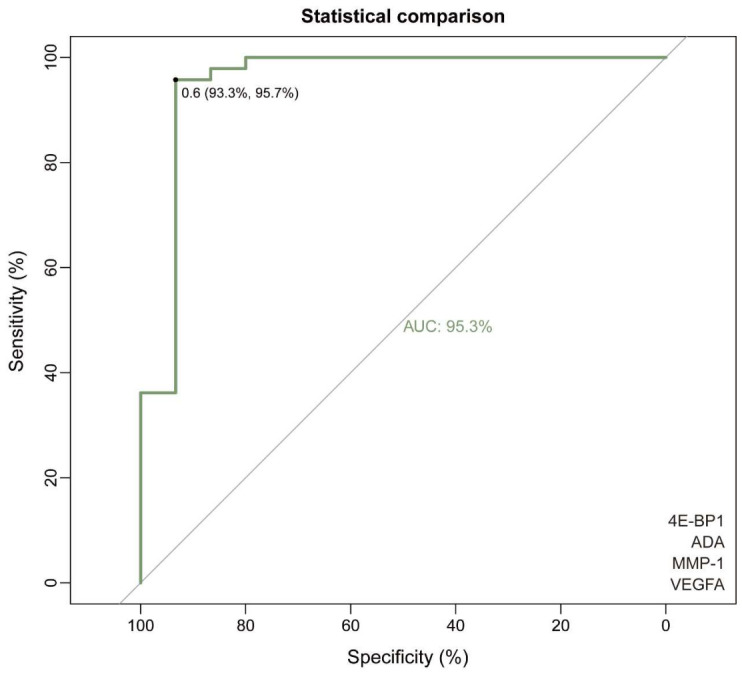
Diagnostic performance of the machine learning-derived multivariable model. The Receiver Operating Characteristic (ROC) curve demonstrates the robust predictive value of the identified four-protein panel (comprising ADA, VEGFA, 4E-BP1, and MMP-1) in distinguishing between patients with DOR and normal controls. The model was validated using a 10-fold cross-validation approach. The AUC value was 95.3%.

To evaluate the predictive performance of these four targets in a clinical setting, a multivariable diagnostic model was constructed. ROC curve analysis demonstrated that the four-protein panel, comprising ADA, VEGFA, 4E-BP1, and MMP-1, had excellent discriminatory power for DOR (Figure 5). Following internal cross-validation, the model yielded an AUC of 0.953 (Supplementary Table S3). Recognizing the significant age discrepancy between the original cohorts (34.36 ± 3.55 years in the DOR group vs. 29.62 ± 3.68 years in the CON group, *p* < 0.001), we conducted an age-matched subgroup analysis to eliminate potential age-related confounding. By selecting a younger subset of DOR patients, the age difference was narrowed (31.63 ± 2.42 vs. 29.62 ± 3.68 years). Re-evaluating the multivariable model on this matched subset via 10-fold cross-validation yielded a robust AUC of 0.901, confirming the signature’s high diagnostic value independent of chronological aging (Supplementary Tables S4 and S5). These findings demonstrate the robust predictive value of the ADA/VEGFA/4E-BP1/MMP-1 signature for the early detection of DOR and the assessment of the follicular microenvironment.

## 4. Discussion

DOR is an increasingly prevalent challenge in contemporary reproductive medicine. It affects approximately 10–24% of women undergoing IVF and is a primary cause of poor reproductive outcomes [17,18]. A major predicament in the clinical management of DOR is the lack of a universal gold-standard diagnostic method [6]. Current diagnostic frameworks predominantly rely on systemic endocrine markers, such as the serum AMH concentration and the basal AFC [19]. However, these indirect peripheral indices primarily reflect the quantitative depletion of the primordial follicle pool, and do not adequately capture the qualitative deterioration of the ovarian microenvironment [20]. FF directly nurtures the developing oocyte and contains a myriad of secreted proteins that mirror early pathophysiological changes, and particularly localized chronic inflammation [21,22,23]. Therefore, the use of FF-derived secreted proteins as auxiliary diagnostic biomarkers represents a highly promising means of bridging the current diagnostic gap [24]. In the present study, using ultra-sensitive Olink targeted proteomics combined with a machine learning pipeline, we successfully identified a candidate four-protein signature in FF, comprising ADA, VEGFA, 4E-BP1, and MMP-1, which has strong potential as a diagnostic indicator of DOR. We hypothesize these four biomarkers may reflect key aspects of follicular decline: immune dysfunction, vascular degeneration, apoptosis, and fibrotic stiffening of the extracellular matrix (ECM). This signature has potential as an auxiliary diagnostic tool and may provide insight into the localized follicular pathogenesis of DOR.

ADA is a key purine metabolic enzyme that catalyzes the irreversible deamination of adenosine to inosine [25]. Physiologically, extracellular adenosine functions as a potent endogenous immunoregulatory molecule, suppressing excessive inflammatory responses and protecting tissues from collateral damage, primarily via the activation of A2A receptors on immune cells [26]. The present proteomic analysis has revealed significant dysregulation of ADA in the FF of patients with DOR, suggesting a disruption of this purinergic homeostatic mechanism. This resulting chronic inflammation may exacerbate oxidative stress in oocytes and follicular somatic cells, ultimately impairing oocyte quality and accelerating follicular atresia [27,28].

Follicular growth and subsequent ovulation are dependent on the cyclical proliferation and dynamic remodeling of the capillary network [29,30]. VEGFA is the master regulator of this angiogenesis, which ensures adequate delivery of oxygen, gonadotropins, and metabolic substrates to the rapidly dividing granulosa cells [31]. The present findings highlight the potential of VEGFA as a core predictive biomarker and underscore the essential role of vascular integrity in the maintenance of the ovarian reserve. Recent advances in reproductive biology have established “ovarian vascular aging” as a hidden driver of declines in female fertility [32]. In the present study, we found higher expression of VEGFA and FSHR in the granular cells of older women, suggesting that the follicular development capacity may decrease because of insufficient blood flow in the aging ovary [32]. In studies of endometriosis, VEGFA expression has been shown to positively correlate with that of HIF-1α and IL33, which jointly promote inflammatory responses [33]. Consistent with this, another study showed that the overexpression of hVEGF165b in mouse ovaries leads to reductions in the size of the ovaries and the number of primordial follicles [34]. The present proteomic analysis has revealed an abnormally high VEGFA concentration in the FF of patients with DOR. This altered angiogenic profile is hypothesized to leave the developing follicles in a state of chronic ischemia and hypoxia. Such deprivation could blunt the responsiveness of granulosa cells to gonadotropins and potentially trigger endoplasmic reticulum stress. This suggests that microvascular degeneration might serve as a critical pathological nexus connecting local inflammation to poor IVF outcomes.

The survival and proliferation of granulosa cells, which provide indispensable metabolic support to the oocyte, are strictly governed by the PI3K/AKT/mTOR signaling cascade [35,36], and 4E-BP1 is a critical downstream repressor of this pathway [37]. Under optimal conditions, active mTOR phosphorylates and inhibits 4E-BP1, liberating eIF4E to initiate cap-dependent mRNA translation and drive robust cell growth [37,38]. In addition, the genetic deletion of 4E-BP1/2 in macrophages diminishes anti-inflammatory effects, potentially triggering the abnormal transcription program of inflammation-related factors [39]. The absence of 4E-BP1 in the DOR follicular microenvironment may lead to abnormal inflammatory responses and dysregulation of the translation program. For highly proliferative granulosa cells, this abnormal translation process and inflammatory response halt cellular growth and critically deplete anti-apoptotic proteins. This apoptotic wave severs the vital bidirectional communication between the granulosa cells and the oocytes, directly precipitating follicular atresia [40].

The ovary is a highly dynamic organ that requires continuous remodeling of its ECM to accommodate the substantial physical expansion of growing follicles. MMP-1, an interstitial collagenase, plays a pivotal role in the maintenance of ECM plasticity by degrading structural collagens (types I, II, and III) [41]. The machine learning approach used in the present study identified MMP-1 as a key component of the DOR signature, shedding light on the critical physical constraints imposed on follicles.

In the state of chronic ovarian inflammation, the delicate physiological balance between MMPs and their tissue inhibitors is severely disrupted [42]. The chronic inflammatory environment triggers an aberrant tissue healing response, leading to the pathological accumulation of cross-linked collagen within the ovarian stroma—a condition recognized as ovarian fibrosis [43]. Dysregulated expression of MMP-1 indicates impaired ECM remodeling [44]. As the ovarian stroma becomes increasingly fibrotic and mechanically stiff, the physical resistance to follicle expansion increases [45]. Therefore, MMP-1 is a biomarker that reflects the transition from an inflammatory microenvironment to irreversible pathological tissue remodeling, wherein fibrotic stiffening is hypothesized to physically restrict and deplete the functional ovarian reserve.

We selected LASSO regression over Ridge regression and Elastic Net to screen potential biomarkers. LASSO offers a distinct advantage in feature selection by applying an L1 penalty, which forces the coefficients of non-contributing proteins to become zero. In contrast, ridge regression retains all the predictors, thereby reducing their clinical interpretability, and Elastic Net often introduces additional complexity. LASSO efficiently identified a refined four-protein signature, and the parsimony of this model is critical for the development of a cost-effective diagnostic tool for DOR.

Despite the robustness of the targeted proteomic profiling and machine learning analyses used in the present study, several limitations warrant consideration. First, the age disparity between the groups is a potential limitation. We focused on characterizing the follicular inflammatory proteome as a molecular fingerprint of DOR, and subsequent studies with age-matched cohorts should be performed to further establish the robustness and independence of the identified signature. Second, although the model demonstrated robust performance and high AUC values during the 10-fold cross-validation, the small sample size and the single-center design remain limitations. Future studies of large-scale, multicenter, and prospective validation cohorts are imperative to confirm the clinical predictive efficacy and prognostic value of the ADA/VEGFA/4E-BP1/MMP-1 model for pregnancy outcomes. Finally, although our model demonstrated strong statistical associations, they are insufficient to confirm causality. Therefore, further basic research using in vitro assays and in vivo models is required to elucidate the precise molecular mechanisms by which these four targets drive ovarian inflammaging, ischemia, and ECM remodeling. Nevertheless, the present study provides supporting evidence that an FF-derived inflammatory signature holds tremendous potential as both a candidate diagnostic biomarker and a therapeutic target for the preservation of fertility in patients with DOR.

## 5. Conclusions

We have identified four protein molecules (ADA, VEGFA, 4E-BP1, and MMP-1) that are significantly associated with DOR status in the follicular microenvironment. Furthermore, using a multivariate machine learning approach, we have constructed a candidate auxiliary diagnostic model for DOR. Given the exploratory nature of this study, the findings warrant further validation in large-scale, independent cohorts to confirm their clinical utility and prognostic value.

## Figures and Tables

**Table 1:** Baseline characteristics of the participants (n = 88).

Characteristics	DOR (67)	CON (21)	*p*
Age (years)	34.36 ± 3.55	29.62 ± 3.68	<0.001
BMI (kg/m^2^)	22.30 ± 2.52	22.74 ± 2.77	0.498
AMH (ng/mL)	0.97 ± 0.46	3.91 ± 1.17	<0.001
TSH (mIU/L)	2.26 ± 1.11	2.29 ± 0.92	0.927
FSH (IU/L)	10.21 ± 6.64	8.06 ± 3.24	0.215
LH (IU/L)	4.92 ± 2.35	6.95 ± 7.18	0.064
E_2_ (pg/mL)	37.45 ± 19.27	37.06 ± 22.18	0.938
T (nmol/L)	4.35 ± 15.32	0.81 ± 0.52	0.393
AFC	4.79 ± 1.69	11.52 ± 4.21	<0.001
Number of cycles	3.31 ± 2.66	1.24 ± 0.63	0.001
Normal fertilized oocytes	10.61 ± 8.01	16.43 ± 7.50	0.005
Transferable embryos	3.69 ± 2.17	5.62 ± 2.22	0.001
ART			<0.001
IVF	41 (61.19%)	11 (52.38%)	
ICSI	17 (25.37%)	10 (47.62%)	
PGTA	9 (13.43%)	0	
Pregnancy			<0.001
Yes	24 (35.82%)	18 (85.71%)	
No	43 (64.18%)	3 (14.29%)	

DOR: diminished ovarian reserve; AMH: anti-Müllerian hormone; FSH: follicle-stimulating hormone; LH: luteinizing hormone; TSH: thyroid-stimulating hormone; E_2_: estradiol; T: testosterone; AFC: antral follicle count. ART: assisted reproductive technique; IVF: in vitro fertilization; ICSI: intracytoplasmic sperm injection; PGT-A: preimplantation genetic testing for aneuploidy. *p* < 0.05 indicates statistical significance (Student’s *t*-test, Mann–Whitney U test, or Chi-square test).

**Table 2:** Differentially expressed inflammatory proteins in the FF of the DOR and CON groups.

OLINK ID	Protein Symbol	Name	ΔNPX (Log_2_FC)	Trend	*p*-Value
OID00536	4E-BP1	Eukaryotic translation initiation factor 4E-binding protein 1	−1.017	DOWN	<0.001
OID00534	CXCL6	C-X-C motif chemokine ligand 6	−0.838	DOWN	<0.001
OID00558	STAMBP	STAM-binding protein	−0.622	DOWN	<0.001
OID00560	ADA	Adenosine deaminase	−0.446	DOWN	<0.001
OID00503	TGF-alpha	Transforming growth factor alpha	−0.663	DOWN	<0.001
OID00538	SIRT2	Sirtuin 2	−0.717	DOWN	<0.001
OID00541	EN-RAGE	S100 calcium-binding protein A12	−1.275	DOWN	0.001
OID00521	TRANCE	Tumor necrosis factor superfamily member 11	−0.505	DOWN	0.001
OID00506	TNFSF14	Tumor necrosis factor superfamily member 14	−0.573	DOWN	0.002
OID00475	GDNF	Glial cell line-derived neurotrophic factor	−0.553	DOWN	0.002
OID00562	CSF-1	Colony-stimulating factor 1	−0.208	DOWN	0.004
OID00542	CD40	CD40 molecule, TNF receptor superfamily member 5	−0.317	DOWN	0.004
OID00531	CD5	CD5 molecule	−0.324	DOWN	0.005
OID00472	VEGFA	Vascular endothelial growth factor A	0.343	UP	0.006
OID00530	CCL23	C-C motif chemokine ligand 23	−0.427	DOWN	0.008
OID00488	TRAIL	Tumor necrosis factor superfamily member 10	−0.265	DOWN	0.009
OID00496	CXCL1	C-X-C motif chemokine ligand 1	−0.614	DOWN	0.009
OID00515	IL-10RB	Interleukin 10 receptor subunit beta	−0.283	DOWN	0.012
OID00494	OSM	Oncostatin M	−0.705	DOWN	0.014
OID00511	LIF-R	LIF receptor alpha	−0.192	DOWN	0.016
OID00497	TSLP	Thymic stromal lymphopoietin	0.702	UP	0.021
OID00533	Flt3L	FMS-like tyrosine kinase 3 ligand	−0.306	DOWN	0.021
OID00555	TWEAK	Tumor necrosis factor superfamily member 12	−0.247	DOWN	0.022
OID00477	CD244	CD244 molecule, natural killer cell receptor 2B4	−0.211	DOWN	0.024
OID00480	LAP TGF-beta-1	Latency-associated peptide transforming growth factor beta-1	−0.200	DOWN	0.029
OID01213	DNER	Delta and Notch-like epidermal growth factor-related receptor	−0.252	DOWN	0.029
OID00508	IL-10RA	Interleukin 10 receptor subunit alpha	0.110	UP	0.031
OID00510	MMP-1	Matrix metalloproteinase 1	0.436	UP	0.032
OID00501	IL18	Interleukin 18	−0.361	DOWN	0.035
OID05124	CD8A	CD8a molecule	−0.333	DOWN	0.036
OID00517	IL-18R1	Interleukin 18 receptor 1	−0.302	DOWN	0.037
OID00528	IL10	Interleukin 10	−0.600	DOWN	0.039
OID00556	CCL20	C-C motif chemokine ligand 20	−0.153	DOWN	0.039
OID00550	CASP-8	Caspase 8	−0.348	DOWN	0.040
OID00485	IL-17A	Interleukin 17A	−0.247	DOWN	0.047

ΔNPX (Log_2_FC) represents the difference in mean NPX values (DOR group minus Control group). ‘Up’ indicates higher expression in the DOR group.

**Table 3:** Results of the conditional logistic regression analysis of the relationships between the risk factors and DOR.

	Univariable Analysis		Multivariable Logistic Regression	
Variables	*p*	Exp(B) (95% CI)	*p*	Exp(B) (95% CI)
Age (years)	0.010	0.81 (0.69~−0.95)	0.020	0.82 (0.69~0.97)
BMI (kg/m^2^)	0.786	1.03 (0.84~1.26)		
AMH (ng/mL)	0.129	2.38 (0.78~7.32)		
TSH (mIU/L)	0.940	1.02 (0.64~1.62)		
FSH (IU/L)	0.346	0.95 (0.86~1.06)		
LH (IU/L)	0.308	1.12 (0.90~1.41)		
E_2_ (pg/mL)	0.047	0.97 (0.95~1.00)	0.043	0.96 (0.93~1.00)
T (nmol/L)	0.522	0.95 (0.81~1.11)		
AFC	0.136	1.27 (0.93~1.75)		
Number of cycles	0.038	0.708 (0.51~−0.98)	0.038	0.67 (0.46~0.98)
Normal fertilized oocytes	0.440	1.03 (0.96~1.09)		
Transferable embryos	0.951	1.01 (0.80~1.27)		
ART	0.623			

## Data Availability

The original contributions presented in this study are included in the Supplementary Material. Further inquiries can be directed to the corresponding authors.

## Supplementary Materials

The following supporting information can be downloaded at: https://www.mdpi.com/article/10.3390/jcm15114072/s1. Figure S1: RCS models of the relationships between follicular fluid DEPs and clinical indices; Table S1: Baseline demographic and clinical characteristics of all study participants; Table S2: Olink raw count table; Table S3: AUC values of training and test sets across 10-fold cross-validation.; Table S4: Baseline demographic and clinical characteristics of participants after age matching.; Table S5: AUC values of training and test sets across 10-fold cross-validation after age matching.
