# Supplementary figures and images for: Identification of Inflammatory Markers for the Prediction and Diagnosis of Diminished Ovarian Reserve Using Olink Targeted Proteomics
Source: J Clin Med. 2026 May 25;15(11):4072. doi: 10.3390/jcm15114072 (PMC13257799; doi:10.3390/jcm15114072)

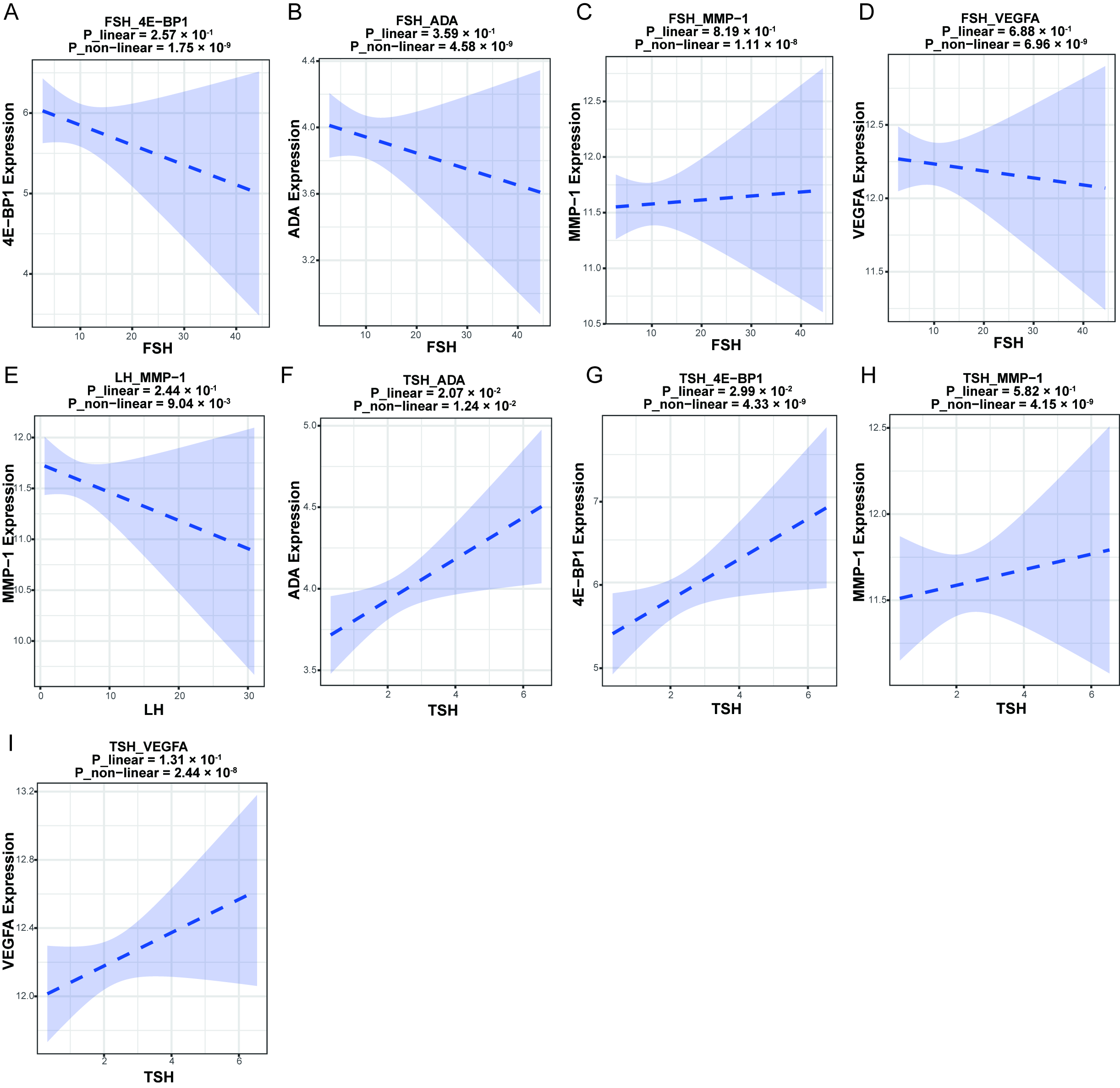

Supplement: Supplementary file 1 [file jcm-15-04072-s001.zip › Supplementary Figure S1.tif]
